# Lessons learned from the pre-implementation phase of a patient navigation intervention to increase patient portal enrollment in Federally Qualified Health Centers

**DOI:** 10.3389/frhs.2025.1624032

**Published:** 2025-09-30

**Authors:** Alicia K. Matthews, Safa Elkefi, Maureen George, Andrea Cassells, Jonathan N. Tobin

**Affiliations:** ^1^ School of Nursing, Columbia University, New York, NY, United States; ^2^ School of System Sciences & Industrial Engineering, Watson College of Engineering, Binghamton University, Vestal, NY, United States; ^3^ Clinical Directors Network (CDN), New York, NY, United States; ^4^ The Rockefeller University Center for Clinical and Translational Science, New York, NY, United States

**Keywords:** patient navigators, patient portals, federally qualified health centers, electronic health records, pre-implementation interviews

## Abstract

**Objectives:**

To describe the pre-implementation phase of a patient navigator-led intervention to increase patient portal enrollment among adults receiving care within Federally Qualified Health Centers (FQHCs) in New York City.

**Methods:**

We conducted semi-structured in-depth interviews with fourteen key stakeholders (clinicians, nurses, patient navigators, and practice staff) in three FQHCs. Using the Exploration, Preparation, Implementation, and Sustainment (EPIS) framework as a guide, the interviews focused on current patient portal education and enrollment procedures, establishing the workflow for the new patient navigator-led enrollment intervention, co-creation of low-health literacy educational materials, and identifying potential challenges and mitigation strategies. Thematic analysis was conducted to inform the development of a standardized patient portal enrollment protocol.

**Results:**

Findings revealed significant variability in support and educational procedures across the three FQHC locations. Strategies that emerged as potentially effective for integrating patient navigators into the center workflow included scheduling navigators during peak hours (Mondays to Thursdays, 10 AM to 4 PM) and positioning them in high-traffic areas such as waiting rooms. Customizing educational materials to meet linguistic and cultural needs was important for improving accessibility and relevance. Providing navigators with access to the appointment scheduling and Electronic Health Records (EHR) systems was viewed as enabling real-time identification and engagement of eligible patients, reducing missed enrollment opportunities. Proactive engagement methods, including in-lobby interactions, were viewed as essential in fostering sustained portal usage. Addressing technological barriers and language challenges through multilingual resources and hands-on demonstrations was also described as creating a more inclusive environment.

**Conclusions:**

The study results have implications for implementing and evaluating a patient navigator-led intervention to increase patient portal enrollment among patients in FQHCs. Hiring and training dedicated navigators, customizing educational materials, and integrating navigators into the practice's workflow are key strategies for improving the adoption of this intervention. The findings provide a foundation for future research to evaluate the effectiveness, sustainability and scalability of the intervention approach across diverse healthcare settings.

## 1 Introduction

Federally qualified health centers (FQHCs) are community practices that provide comprehensive primary and preventive health care to individuals in underserved areas (1). As of July 2024, approximately 1,400 FQHCs operate across more than 19,000 sites in the United States and its territories, serving over 30.5 million patients, 91% of whom have incomes below 200% of the federal poverty level (1).

Many FQHCs are expanding health-promotion activities, including cancer prevention and control (2), by leveraging electronic health records, linked patient portals, secure online tools for accessing personal records, messaging providers, viewing test results, scheduling, and managing prescriptions and billing (3–5) as well as through in-person and telephone patient navigation and prevention care management.

Patient portals have the potential to support individual and population-based outreach strategies for increasing awareness and engagement in health promotion initiatives (5–7). For example, a study by McCleary et al. used patient portals to improve patient engagement in care in ambulatory oncology practices (8). Another study by Matthews et al. was designed to use patient portals to provide smoking cessation advice and automated referral to smoking cessation services among patients in FQHCs (9). Asynchronous physician-assisted smoking cessation interventions have also been conducted using electronic portal messaging as per a study presented by Erdmann et al. (10). Several systematic reviews have concluded that patient portal interventions can lead to improvements in knowledge, self-confidence, patient decision-making, treatment adherence, the quality of the patient-provider relationship, and the use of preventative services (11, 12). Despite the established benefits of patient portal-led interventions, patient portal enrollment remains low, impacting the potential success of such interventions (13, 14).

Research has shown that although engagement with patient portal usage has increased over the years, it remains low among the general population (15). As of 2020, approximately forty percent of U.S. adults were enrolled in a patient portal (16). Vulnerable populations often demonstrate lower health literacy and experience significant barriers to care (17). Portal features such as messaging, online education, and automatic medication refills might increase convenience, improve health literacy, and overcome at least some barriers to care, thereby reducing health inequities (17, 18). Unfortunately, a study by Grossman et al. stated in 2019 that more than 100 studies show substantial health-equity–relevant disparities in portal use among older adults, racial minorities, as well as people with low socioeconomic status (18–20). Relatively low portal use in vulnerable populations, such as older populations and people who don't have access to technology, may lead to intervention-generated inequity (21). Thus, developing, implementing, and evaluating strategies to reduce disparities in portal usage remains critical to ensuring portals benefit all populations (18). Similarly, increasing the uptake of electronic health record (EHR)-linked patient portals in FQHC settings could help transform prevention and control strategies for cancer and other chronic diseases (9).

Low enrollment can be linked to limited awareness of the availability and benefits of patient portal enrollment and the absence of staff support for assisting patients in portal enrollment and usage (6). Studies have shown that individually focused interventions have the most evidence for increasing portal use in vulnerable populations (18, 22–25). Patients are more likely to enroll and use their portals if encouraged by their healthcare providers and patient navigators and offered assistance (26, 27).

This study summarizes the qualitative findings from a series of pre-implementation interviews to guide the development and execution of a patient navigator-led campaign to enroll patients into the FQHCs patient portal. The campaign focused on increasing enrollment among low-income patients associated with three Federally Qualified Health Centers (FQHCs) in New York City. The results of the interviews were used to establish the materials and implementation workflow for a future patient navigator-led strategy for increasing patient portal enrollment among patients in FQHC settings.

## 2 Methods

### 2.1 Study design & context

**Figure 1:**
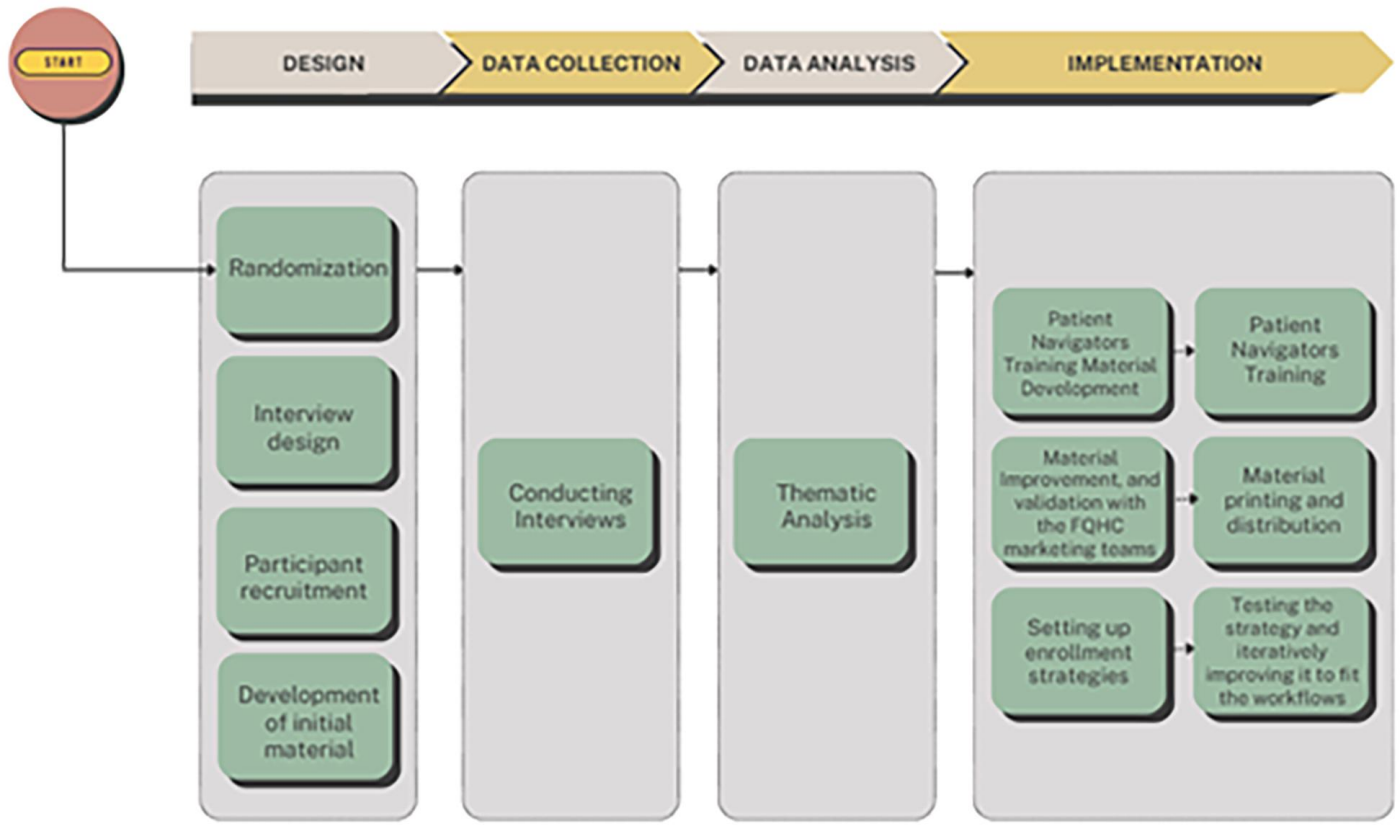
Summary of the pre-implementation phase process.

This study was conducted in three FQHCs in the New York area. This manuscript focuses on the pre-implementation qualitative phase of a broader randomized controlled trial (RCT). While the larger RCT aims to (1) promote enrollment in patient portals across the three FQHCs, and (2) compare different EHR-based smoking cessation referral strategies across them, the present paper reports only on the qualitative component conducted prior to the portal enrollment initiative to inform implementation planning (see Figure 1). The study received IRB approval from Columbia University under the number IRB-AAAU7659.

### 2.2 Study settings & randomized trial overview

This study was conducted in three FQHCs affiliated with Clinical Directors Network, Inc. (CDN). CDN is a not-for-profit clinician membership organization, practice-based research network (PBRN) and AHRQ-designated Center of Excellence (P30) for Practice-based Research and Learning. CDN was founded as a clinician training organization to provide peer-initiated activities for clinicians practicing in low-income, minority, and other underserved communities (28). CDN's overall goal is to translate clinical research into clinical practice for the enhancement of health equity and improvement of public health (28).

Our project aimed to compare the effectiveness of two strategies to support enrollment in patient portals: education and patient navigation vs. patient navigation only. Three sites were randomized. The first center was the control site, where only patient education material was shared. The two intervention centers were provided with educational materials and patient navigation support.

### 2.3 Participants and recruitment

As part of the pre-implementation phase (prior to randomization), we conducted interviews with stakeholders affiliated with the three participating FQHCs. Participants were recruited using convenience sampling. FQHC leadership distributed an open invitation to all clinical and administrative staff via internal email. Staff who found the study relevant and whose schedules permitted voluntarily contacted the research team to express interest in participating.

A total of 14 participants were interviewed, including 2 health information technology specialists, 2 nurses, 3 front desk assistants, 2 medical assistants, 2 health coaches, 2 clinical operations managers, and 1 medical director. After confirming interest, interviews were scheduled and conducted via Zoom. At the beginning of each interview, the study team reiterated the purpose of the study, explained the voluntary nature of participation, and obtained oral consent, including permission to audio-record the session. Interviews lasted between 25 and 40 min. Participants received a $50 gift card as a token of appreciation.

### 2.4 Interview procedures & guide development

Interviews focused mainly on gathering information to (1) learn about the centers' existing processes to support patient portal enrollment, (2) co-develop patient engagement materials, and (3) establish the workflow of patient navigation implementation. The interview guide was developed following the EPIS framework (Exploration, Preparation, Implementation, and Sustainment) (29). EPIS includes four distinct phases: (1) Exploration: assessing current practices and identifying barriers and needs; (2) Preparation: planning and adapting strategies to the setting; (3) Implementation: executing and monitoring the intervention; and (4) Sustainment: promoting long-term integration into practice (30).

The framework also accounts for inner and outer contextual factors, innovation characteristics, and bridging elements between systems and organizations. Correspondingly, the interview guide included questions such as (see Appendix 1 for all the questions):

- Exploration (e.g., *Please describe the current center's procedures for educating patients about patient portals in general. Are you educating them about how important they are to them?)*
- Preparation (e.g., *Are there any points that you think we should be aware of while designing our patient navigation for portal enrollment to align with the staff's missions and jobs*?)
- Implementation (e.g., *From your perspective, what resource allocation or streamlining strategies could make the implementation of patient navigation for portal enrollment smoother and more efficient?)*
- Sustainment (e.g., *It may be easier to get patients to enroll than to ensure they continuously use the patient portal. What strategies or adjustments can we implement to enhance the long-term sustainability of patient navigation for portal enrollment and the continuous use of these portals?)*

Prior to developing the patient engagement materials, the study team held meetings with Clinical Directors Network (CDN), who shared examples of existing materials used at the centers. CDN also organized sessions with the FQHCs' IT teams, who provided demonstrations of the patient portal systems, including walkthroughs of navigation workflows and currently enabled features. These sessions, combined with the interview findings, informed the creation of draft educational materials. Once drafted, the materials were shared with center leadership for review. Written feedback was then collected through follow-up emails and meetings coordinated by CDN to finalize the materials for implementation.

### 2.5 Data analysis

All interviews were audio-recorded, transcribed locally, and de-identified to ensure confidentiality. The analysis followed a qualitative thematic analysis using a hybrid deductive-inductive approach, guided by the EPIS framework and Braun and Clarke's (2012) six-phase method for thematic analysis (31). These six phases include: (1) familiarization with the data, (2) generation of initial codes, (3) searching for themes, (4) reviewing themes, (5) defining and naming themes, and (6) producing the report.

Two coders independently analyzed the transcripts using a hybrid deductive-inductive coding strategy. An initial draft codebook was developed based on the interview guide, EPIS domains (Exploration, Preparation, Implementation, Sustainment), and key areas of inquiry relevant to the implementation goals of the study. After reviewing both transcripts and audio recordings, the coders expanded the codebook by identifying emergent themes not captured by the initial codes.

Iterative rounds of coding and discussion were conducted to refine the code definitions and structure. Coding was completed using Microsoft Excel, which was also used to document coding decisions, organize excerpts, and track the development of themes across interviews. Discrepancies in coding were addressed through a consensus-building process that involved repeated review and discussion of the transcript sections in question. Thematic saturation was determined by the recurrence of themes across sites and interviews.

Crosscutting themes were synthesized from the final code structure, capturing shared patterns as well as site-specific insights. The final themes were then used to inform the design of the navigator workflow, the development of tailored educational materials, and the implementation planning document. These themes included: (1) feedback about current enrollment procedures, (2) suggested improvements to the process, (3) optimal timing and location for enrollment support, (4) promotion strategies, (5) navigator challenges, (6) potential solutions, and (7) long-term sustainability considerations.

## 3 Results

Fourteen interviews were conducted with key stakeholders at each of three different FQHCs' (*N* = 5 at Site 1, *N* = 5 at Site 2, and *N* = 4 at Site 3) see Table 1. The interviews yielded feedback and information that helped prepare for the implementation of patient navigation and education interventions to support patient enrollment in the three FQHCs. The intervention centers use Healow as a portal, and the control center uses NextGen.

**Table 1:** Demographic characteristics of the interviewees.

Characteristics	Categories	Site 1	Site 2	Site 3	Total
Gender	Female	4	3	4	11
	Male	1	2	0	3
Race/Ethnicity	White	1	1	0	2
	Hispanic	1	0	0	1
	Black	1	1	1	3
	Asian	1	1	0	2
	Other or unknown	1	2	3	6
	Total	5	5	4	14

The participants viewed the patient portal enrollment as a “*very important step in the quality improvement efforts*” of the centers, mainly since only one of the centers reported having a patient navigator to help with portal enrollment. The centers provided the needed support (staff to answer the questions, support from the nurses in the implementation phase, etc.).

### 3.1 Description of current enrollment and educational procedures at each FQHC location

#### 3.1.1 Variability in support and lack of dedicated education

Based on the information collected, it was noted that the level of support and responsibility for enrollment varies across centers. One center has a dedicated staff member (a patient navigator):

“… while the patients are in a waiting area, we do have a navigator that comes around and asks if any of them would be interested in downloading and signing up for the Healow app.” [Site 2]

Others rely on front desk personnel or providers. Although the front desk personnel and providers do their best to provide support if requested by patients, the workload and time constraints, in addition to the limited training, lead to gaps in their ability to assist patients effectively.

“Yeah. I don't think there's actually somebody assigned to just help the patient with the portal. It's all on the front desk, and the front desk is very overburdened with other tasks that they need to do.” [Site 2]

Sometimes, staff members, including front desk personnel, lack sufficient knowledge about the patient portal, which results in their inability to provide comprehensive assistance to patients.

“… The leadership team needs to be able to train the navigator appropriately on the portal. It's all new for us… We are still learning as well.” [Site 1]

#### 3.1.2 Limited patient support

Interviews with stakeholders across each of the three different FQHCs revealed that the assistance provided to patients also differed. In some, it is limited to basic technical tasks like downloading the application or resetting the password without further guidance on using the portal.

“… we help them with what they need help with because sometimes the patient has a different problem with the portal, like sometimes they forget the password or they lock themselves in, or you set them up, or they are brand new.” [Site 3]

While in other centers, patients are more educated on the benefits of portal usage as well:

“… we let any new patients coming in for an appointment know that we do have a patient portal, and we tell them, like, what benefits it is to sign up for the patient portal, and then we also provide them with the informational flyer that we have letting them know that if they do decide to sign up for the portal.” [Site 1]

#### 3.1.3 Reliance on patient initiative and inconsistent educational efforts

Several participants noted a lack of standardization in patient education. Both of the intervention centers have Healow flyers showing the app's essential services, which are available upon request. However, although the material is available, its visibility and placement are not always optimal. Additionally, patients are relied on to inquire or follow up on the information provided. Staff may ask if patients are interested in enrolling but often do not actively encourage or guide them through the process.

“We notify patients that we have a Healow patient portal. We have them sign a consent to be web-enabled to access the portal, then we print them a one-pager, which kinda indicates how they activate the portal, etc.” [Site 3]

“(when asked about available informative posters)… So far, I don't see any. I'm usually in the office here and don't see anything hanging around.”[Site 2]

“I think it's part of their sign-in process where they have something they need to sign if they want portal access. Usually, if they want portal access, the front desk gives them information as to the password or how to do it. I don't think anybody's there educating the patient on how important it is.”[Site 3]

### 3.2 Possible recommendations for changes in the enrollment and educational procedures suggested

The interviews informed us of the days and times it would be best to have a patient navigator in place. For instance, Mondays to Thursdays are the busiest days, and more patients would be in the centers between 10 AM and 4 PM, which would give the navigators a higher chance of finding eligible patients to enroll. In addition, waiting rooms were the areas that navigators should stay in to be close to the patients while waiting for their appointments.

#### 3.2.1 Customize the material to the needs of each center

Feedback about the flyers and posters was also received, and revisions were made to improve the design (content and format) and flow. Three separate versions (one per center) of the material (in English and Spanish) were then developed and updated. Each educational and outreach material was customized to the center's needs and populations (see Appendix 1 for an example). The material was then shared with the marketing teams of the centers for approval before being printed for sharing with patients and posting in different areas of the three sites.

#### 3.2.2 Identify eligible patients without missing walk-ins

We also presented the intended patient navigation and education process to the practice staff at the participating sites and received their feedback on improving it. We intended for a patient navigator to be in the center to support patients in enrollment and education. The navigator would have access to printed lists of the upcoming patients daily, and as patients present for care, they will offer them support.

While printed lists were appreciated, it was advised that navigators be given direct access to the appointment scheduling and electronic health records (EHR) systems to identify eligible patients before approaching them. Navigators also used the EHR to ascertain if a patient had been presented for care and where they were in the queue without disrupting the workflows at the sites.

“So, then that would be perfect because then that means, if they have access to the EHR, once the patients arrive, the navigator will be able to see and will be able to grab them as they are waiting for the nurse or while they are waiting for the providers and in that time they can just update the status to let the rest of the staff know that they have the patient and they're assisting them with enrollment.”[Site 1]

This solution was also suggested to avoid missing unscheduled patients and walk-ins.

#### 3.2.3 Early engagement with the patients and pre- and post-appointment communication

Additionally, it was suggested that patients be approached when waiting in the lobby before their visits to avoid missing them due to a lack of time.

“Some patients are in a rush, and they don't want to talk sometimes like they're, like, like they just wanna meet their doctor and go home.” [Site 2]

One of the participants also suggested that contacting the patients one day before their appointments and informing them that a study is happening could encourage them to arrive early and expect the navigators to help them. Additionally, following up with them after their interactions could reinforce the chances that the portal will be used.

“Maybe there is some telephone outreach the navigator can conduct one day before the appointments to inform patients they will be approaching them.” [Site 1]

“I don't know if it'll be too much for the navigator to call the patient after they are enrolled to say, ‘Hey, I assisted you with enrollment on x,y and z date. Did you get a chance to log in since then?’” [Site 3]

#### 3.2.4 Coordinate with other staff members

Furthermore, it was advised that front desk staff and medical assistants work closely to identify eligible and interested patients and direct them to the navigator without disrupting regular front desk operations. It was also advised to ask providers to check if the navigators approached patients, and if not, they could make a “warm handoff” to them as needed after the visits.

“I think it's a team effort. It makes sense that everyone from the team, from the front desk, the nurses who triage them, the doctors who see them, and even other staff… Just everybody is aware that this is something we have now. Maybe navigators can have a conversation with the front desk staff to say, ‘Hey, I'm looking to speak with this… Can you guys just let me know when they've arrived so that I can touch base?'” [Site 3]

### 3.3 Potential challenges to patient portal enrollment

We obtained the following results when asked about the possible challenges participants think navigators could face.

#### 3.3.1 Patient reluctance and language barriers

First, it was expected that patients could resist enrolling and using portals due to discomfort with technology. Second, they may have some issues remembering passwords or feeling overwhelmed by the information shared with them. Thus, we added a section on the patient portal flyers where the patient's credentials could be written down for them when supported by the navigator. Additionally, practice staff shed light on the difference in language preferences among the patient communities in the area. Thus, the material was developed in the two most spoken languages in the centers, English and Spanish, with the possibility of being extended to more languages per the navigators' capabilities and the patients' needs.

#### 3.3.2 Operational coordination

Furthermore, interviewees expected that navigators may be overwhelmed with the different tasks, especially during rush hours. Thus, it was suggested that they coordinate with the other staff members to effectively approach as many patients as possible. Navigators also need to manage patient interactions without overwhelming the medical and front desk staff, which is where having access to the EHR systems may help as well.

#### 3.3.3 Patient frustration and engagement issues

Based on the feedback, we also noted that capturing and retaining patients' attention can be difficult, mainly when they are preoccupied with other health-related concerns. Thus, navigators should consider that patients may get frustrated easily and respect that when approaching the patients and offering them support.

**Figure 2:**
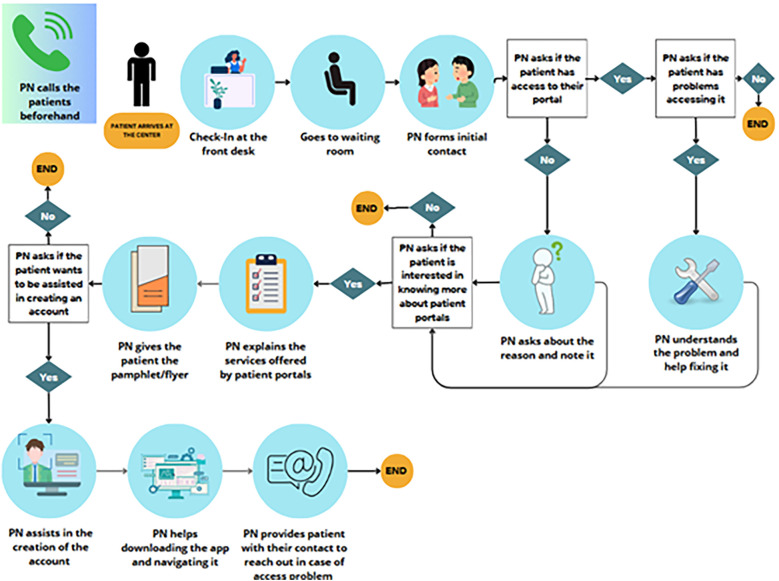
Patient navigation process flow diagram for one of the sites.

The comments received were synthesized and implemented in the navigation strategy setup. Each center was then provided with the education material, training on the portal and the workflow, a handbook on the different steps and best practices, and a process document that summarizes the steps to be taken by each navigator (see Figure 2) and the significant recommendations given.

### 3.4 The patient navigation process flow

As shown in Figure 2, the patient navigator will try to contact the patients one day before their appointment. Once they arrive at the center, the patient checks in at the front desk and is oriented by the staff to sit in the waiting room where they expect the patient to be patient navigators. One of the navigators will then contact the patient and identify their eligibility. Eligible patients are adults who speak English or Spanish. The navigator will then ask if the patient has access to their portals. If yes, they will ask about usage and offer any support with the existing account as needed. Otherwise, the patient will be asked why they do not have access and whether they are interested in knowing more about it. If accepted, the navigator will start by sharing the flyer with the patient and using it as educational material to support their discussion on the benefits of the portal and how to navigate it. After educating them, the navigator will offer the patient their support in account creation. If the patient agrees, the patient will also be supported with the app downloading and initial setup. Ultimately, the patient will be offered a follow-up call to assist them if they have trouble accessing or navigating the application. All the steps and outcomes will be documented by the Patient Navigator in RedCap.

### 3.5 Promoting enrollment in and substantial use of portals

Although it is attractive for patients, we acknowledged that they may lack motivation to enroll in portals and to continue using them. Thus, we asked our participants about their opinions on making this process enjoyable and motivating their sustainability in portal usage.

#### 3.5.1 Amplifying information

Navigators were advised to emphasize the portal's convenience, allowing users to access several forms and lab results without going to the center.

“We should let them know that we can publish forms via the portal, that they can schedule appointments via the Healow app, they can relay messages to their providers via the portal, they can see their labs.” [Site 3]

By highlighting how much time- and effort-saving portal access can be, patients are expected to understand its functionality better and find it more appealing.

#### 3.5.2 Simplifying the process

In addition, the participants thought that navigators should use visual aids when showing the patients how to use the portals to help them better imagine how the navigation should be. For that, we added more visuals to the flyers. We also provided the navigators with digital tablets to help them access the portals when talking to patients and to demonstrate and simulate the usage and access to the different features.

#### 3.5.3 Engagement and continuous support

Some team members also recommended that we continuously engage patients through regular updates, reminders, and frequent follow-ups. Continuous support, such as chat or in-person assistance, can address patients' immediate questions or issues while using the portal.

#### 3.5.4 Visibility and promotion

Visible signage and informative material in common areas, including multilingual support, were advised to promote portal usage. Furthermore, social media and other promotion strategies were encouraged to emphasize the portal's practical functionalities, making it more appealing.

#### 3.5.5 Long-term education and feedback

Finally, the participants advised us to gather patient feedback regularly to understand why they might stop using the portal and address those issues. Providing continuous education initiatives on the benefits of patient portals to patients and their family members could also help ensure patients' sustainable interest in portals.

## 4 Discussion

This study describes the implementation processes associated with developing a patient navigator-led intervention to increase the enrollment of low-income patients seen in three FQHCs in New York in the health system–supported and electronic health record–linked patient portals. The feedback collected from the 14 participants helped us gather feedback about the current enrollment and educational procedures and develop new methods for our implementation phase. It also helped us anticipate some challenges we may face in the implementation phase.

### 4.1 Feedback about the current patient portal enrollment and education procedures

The findings revealed significant variability in support and educational procedures across different centers. While one center employed dedicated patient navigators, others relied on front desk personnel or providers who faced challenges such as high workloads, time constraints, and limited training. Additionally, patient support was inconsistent, often restricted to essential technical assistance without comprehensive guidance.

Educational efforts lacked standardization, leading to reliance on patient initiative and suboptimal information dissemination. The variability in support structures suggests that dedicated staff, such as patient navigators, is crucial in facilitating effective patient portal enrollment and usage. Centers lacking such dedicated resources may struggle to provide comprehensive assistance due to overstretched front desk personnel and providers primarily focused on clinical duties. This inconsistency can lead to fragmented patient experiences, where some patients receive thorough guidance while others receive minimal support, potentially affecting their engagement with the patient portal.

Consistent with prior research (27, 32), our study underscores the importance of dedicated support staff in enhancing patient engagement with digital health tools, especially patient portals. Previous studies have shown that when patients receive comprehensive education and support, especially from patient navigators, they are more likely to engage actively with digital health tools, which would improve enrollment rates and patient satisfaction (27). However, unlike some studies that report minimal variability across centers, our findings indicate a substantial disparity, possibly reflecting differences in resource allocation and institutional priorities. Additionally, the observed reliance on patient initiative in some centers contrasts with some literature emphasizing proactive staff engagement as a critical facilitator for patient portal success (33). The inconsistency in educational efforts echoes concerns raised in earlier studies about the lack of standardized patient education protocols, which can lead to variable patient experiences and outcomes (34, 35).

### 4.2 Enrollment and education procedures suggested

These results helped us develop a manual of procedures that can help guide patient navigators in their efforts to enroll patients without disturbing the current workflows. The efforts implemented covered several strategic areas, including optimal scheduling and placement of patient navigators, customization of educational materials, efficient patient identification processes, early patient engagement, and effective coordination with other staff members. The interviews revealed that scheduling patient navigators during peak hours (Mondays to Thursdays, 10 AM to 4 PM) and positioning them in waiting rooms significantly increased the likelihood of enrolling eligible patients. Early engagement strategies, such as pre-appointment communications and in-lobby interactions, emphasize the importance of timing in patient support. By informing patients about the study and portal benefits before their visits, centers can foster a proactive mindset, allowing more time for consideration and decision-making, encouraging timely enrollment and sustained portal usage.

The strategic deployment of patient navigators during peak hours and their placement in high-traffic areas like waiting rooms suggests that visibility and accessibility are crucial for successful patient enrollment. By being present where patients are most likely to congregate, navigators can proactively engage with patients, reducing reliance on patient initiative and ensuring that support is readily available. The strategic placement and scheduling of navigators to coincide with peak patient flow times and locations have been suggested in earlier studies to maximize engagement (36).

Additionally, customizing educational materials to meet each center's unique and specific population needs, including language-specific versions, indicates the importance of culturally and contextually relevant information in enhancing patient understanding and portal utilization, which resonates with previous literature on the effectiveness of tailored health communication in improving patient outcomes (37). This customization ensures that educational efforts resonate with diverse patient demographics in FQHCs, improving engagement, as shown in previous studies (38, 39).

The study also identified the need for granting patient navigators direct access to EHR systems, which addresses a significant systems-level barrier to promptly identifying eligible patients. Granting navigators access enables them to engage with patients in real-time, including those who walk in without prior appointments, ensuring comprehensive coverage and minimizing missed opportunities for enrollment. This approach may differ from some existing models where navigators rely on printed lists or manual referrals, highlighting an area for potential improvement in workflow integration (40). Finally, effective coordination with front desk staff and medical assistants underscores the necessity of an integrated support system. By leveraging the roles of existing staff members, centers can create a seamless referral process that enhances patient support without overburdening any single group.

### 4.3 Possible challenges to face

In addition to identifying existing gaps in support and education, the study explored potential challenges faced by patient navigators and the strategies implemented to mitigate them. Several anticipated challenges were identified. Patient reluctance and language barriers emerged as significant potential hurdles in patient portal enrollment. Patients' discomfort with technology and difficulty remembering passwords can impede their willingness to use digital health tools. By incorporating credential sections into educational materials and providing multilingual resources, centers have taken proactive steps to alleviate these barriers, fostering a more inclusive and supportive environment. Studies have shown that addressing language barriers through multilingual educational materials improves portal enrollment among diverse patient populations (41). Additionally, operational challenges during peak hours have been identified in previous studies, highlighting the need for streamlined workflows and adequate staffing (42). However, adding credential sections to educational flyers to assist with password management is a novel approach, potentially offering a straightforward solution to a common barrier.

Another challenge identified was operational coordination, particularly during peak hours. This highlighted the need for efficient workflow management to ensure that navigators can reach eligible patients without overwhelming existing staff. The recommendation to grant navigators direct access to Electronic Health Records (EHR) systems facilitates timely identification of eligible patients, including walk-ins and unscheduled visits, thereby enhancing enrollment rates. In addition, we identified patient frustration and engagement problems as possible issues. This stems from patients being preoccupied with other health-related concerns, underscoring the importance of empathetic and respectful interactions. Navigators are encouraged to approach patients thoughtfully, recognizing their emotional states and providing support without adding to their stress.

### 4.4 Promoting enrollment in and substantial use of portals

This study's results shed light on suggestions for improving the continuous use of portals among patients. One of the strategies suggested includes emphasizing the portal's convenience and time-saving benefits. Navigators can communicate the practical benefits that resonate with patients' daily lives, increasing their intrinsic motivation to engage with the portal. These findings are consistent with prior research emphasizing the importance of user-friendly design and continuous support in promoting patient portal adoption (43). Studies have also shown that highlighting the practical benefits of patient portals, such as easy access to medical records and lab results, significantly increases patient engagement (44).

Additionally, the use of visual aids and hands-on demonstrations has been identified as an effective method to reduce technological anxiety and improve portal usability (45). However, the specific emphasis on social media promotion represents an innovative approach that is less extensively covered in existing literature. This strategy may offer new avenues for increasing portal visibility and facilitating patient education interactively and engagingly.

Finally, it was advised to implement long-term feedback mechanisms to enable healthcare centers to continuously adapt their strategies based on patient needs and preferences, ensuring that the portal remains relevant and satisfying to patients' needs. Gathering patient feedback continuously allows healthcare centers to identify and address issues promptly, tailoring support and educational materials to meet patients' preferences better and overcome challenges. This iterative process fosters a patient-centered approach, enhancing the portal's usability and relevance and increasing its sustained use. These findings align with existing literature emphasizing the importance of continuous feedback mechanisms for understanding user experiences and making necessary improvements to enhance portal usability (46).

### 4.5 Implications

Based on the findings presented, several practical implications emerge, highlighting actionable strategies and considerations for healthcare organizations to enhance patient portal enrollment and sustained usage, particularly among low-income populations receiving care in Federally Qualified Health Centers (FQHCs). First, the study underscores the critical role of dedicated patient navigators in facilitating effective patient portal enrollment and usage. Healthcare organizations should consider allocating resources to hire and train dedicated patient navigators. These navigators can provide specialized assistance, ensuring patients receive thorough guidance and support, improving enrollment rates and patient satisfaction.

Standardizing patient education protocols is essential as it ensures that all patients receive uniform information and support, reducing portal enrollment and usage disparities. This can involve creating comprehensive training programs for navigators and other staff members to deliver consistent educational content. In addition, healthcare centers should strategically schedule navigators when patient traffic is highest and place them in visible, accessible locations. This approach maximizes opportunities for patient-navigator interactions, facilitating timely enrollment and support without disrupting existing workflows.

Furthermore, developing culturally and linguistically appropriate educational materials is crucial for addressing diverse patient needs. Customization ensures that information is relevant and understandable, enhancing patient understanding and willingness to engage with the portal. Our findings also highlight a tension between the need for standardization and the importance of personalization in patient education. While standardizing the navigator workflow and core educational materials can promote consistency and make implementation more feasible for staff, the interviews underscored that rigid protocols may not adequately address patients' diverse needs.

Several participants suggested that standardization and customization are not mutually exclusive. Instead, they recommended creating a core protocol with built-in flexibility, such as optional scripts, multilingual materials, or adaptable engagement strategies, allowing navigators to tailor their approach based on each patient's language, health literacy, or comfort with technology. This hybrid approach ensures consistent delivery while allowing for responsiveness at the point of care, thus supporting both implementation fidelity and person-centeredness.

Moreover, integrating navigators into the EHR system workflow can streamline the enrollment process. This integration enables navigators to identify and engage eligible patients promptly, reducing missed opportunities and improving overall enrollment rates. It is also noteworthy that implementing proactive engagement strategies may ensure that patients are informed about the portal and its benefits before their visits. This proactive approach can foster a mindset geared towards timely enrollment and sustained usage, enhancing overall patient engagement. Patient reluctance, technological discomfort, and language barriers were significant hurdles. Providing comprehensive support to address these barriers is essential. This includes offering multilingual educational materials, incorporating password management assistance into educational resources, and conducting hands-on demonstrations to reduce technological anxiety. Such measures create a more inclusive and supportive environment for all patients. Healthcare centers should optimize workflow processes to accommodate navigator activities seamlessly. This might involve redefining roles, enhancing staff collaboration, and ensuring that navigators have the necessary tools, such as digital tablets for real-time enrollment, and support to perform their duties efficiently.

Finally, establishing ongoing feedback loops enables centers to monitor patient experiences, identify emerging issues, and make data-driven adjustments to their enrollment and support strategies. This iterative process ensures the patient portal remains relevant, user-friendly, and aligned with patient expectations and maximizes the potential for sustainability.

Although the findings reflect the experiences of a small number of FQHCs in New York City, the challenges and facilitators identified, such as the need for navigator support, tailored educational materials, and workflow integration, are likely relevant to many safety-net settings. However, variability in structural readiness may influence scalability. Differences in staffing resources, digital health maturity, leadership engagement, and the specific patient populations served could impact the feasibility of implementing similar navigator-led interventions elsewhere. For instance, clinics with limited EHR functionality or no established patient portal may require more foundational investments before such strategies can be adopted.

### 4.6 Limitations

While this study offers important insights into the use of patient navigators and educational strategies to enhance patient portal enrollment among low-income populations in FQHCs, several limitations must be acknowledged. First, this study involved interviews with only 14 stakeholders across three FQHCs in New York. This limited sample size may not capture diverse experiences and perspectives in healthcare settings. Consequently, the findings may not be relevant to other FQHCs and safety net practices with varying structures, patient demographics, or resource levels. Also, the qualitative data reflect only the perspectives of staff and stakeholders; direct input from patients was not included in this phase of the study. Although staff insights offer valuable operational and contextual understanding, patient perspectives could have enriched the findings by shedding light on end-user barriers, motivations, and experiences with portal enrollment. Future work should prioritize incorporating patient voices to strengthen intervention design and ensure alignment with patient needs.

Second, the intervention centers utilized different patient portal systems (Healow), while the control center used NextGen. This variation introduces a potential confounding variable, as differences in portal functionality, user interface, and integration with Electronic Health Records (EHR) systems could impact enrollment and usage rates independently of the navigational and educational interventions, although working across multiple EHRs enhances generalizability.

Third, we gathered feedback from healthcare staff and stakeholders, excluding direct input from patients. As a result, the perspectives of the end-users (patients) on barriers to enrollment and portal usage were not directly captured. Including patient voices in future research could provide a more comprehensive understanding of the challenges and facilitators from the user's standpoint. Finally, this research was conducted during the pre-implementation phase, focusing on training, preliminary results, and lessons learned. Consequently, the study does not assess the long-term effectiveness or sustainability of the implemented strategies. Follow-up studies are necessary to evaluate the impact of patient navigators and educational interventions on patient portal enrollment and usage over time.

## 5 Conclusions

This study supports the integration of patient navigators and standardized educational interventions within FQHCs as effective means to bridge the digital divide and promote health equity. Through stakeholder interviews across three FQHCs, significant gaps in current enrollment and educational practices were identified, notably the variability in support structures and the absence of standardized education protocols. Implementing dedicated patient navigators, strategically scheduled and positioned during peak hours, emerged as a practical approach to enhance patient engagement and streamline enrollment. Additionally, customizing educational materials to address linguistic and cultural needs proved essential in making portal information more accessible and relevant to diverse patient populations. By adopting these strategies, healthcare organizations can enhance patient engagement with digital health tools, ultimately improving health outcomes and reducing disparities among underserved populations. Future research should focus on expanding these initiatives and evaluating their long-term impact to ensure scalability and sustained effectiveness across diverse healthcare settings.

## Data Availability

The raw data supporting the conclusions of this article will be made available by the authors, without undue reservation.

## Appendix 1. Questions used in the interview guide

**Table:**

Domain	Question
Exploration	**Question 1.** Please describe the current clinic procedures for educating patients about patient portals in general. Are you guys educating them about how important they are to them?
	**Question 2.** Please describe the clinic procedures for educating patients about enrolling patients into the patient portal.
Preparation	**Question 3.** Which time (timeframe and days) do you think is best to have PN in the clinics setting?
	**Question 4.** Which location is best for the patient navigators to talk to the patients (lobby, designated space, clinic room)?
	**Question 5.** In terms of the patient navigators' workflow for identifying and approaching eligible patients, we are thinking of getting a daily list of patients and selecting the ones who are not enrolled in the Patient Portals. The printed list could help target the specific patients that need training. What do you think about the process? And how can we make it more secure and efficient?
Implementation (Acceptability, Appropriateness, Feasibility)	**Acceptability:** • **Question 6.** What are your thoughts on how we can enhance the appeal and credibility of the patients' educational patient navigation for portal enrollment promoting patient portal use? • **Question 7.** In your opinion, what aspects of the patient navigation for portal enrollment could be adjusted or focused on to meet better the preferences and expectations of both staff and patients? **Appropriateness:** • **Question 8.** What do you think we should do to make the patient navigation for portal enrollment in alignment with the patient's needs and preferences? • **Question 9.** Are there any points that you think we should be aware of while designing our patient navigation for portal enrollment to align with the staff's missions and jobs? • **Question 10.** Are there any creative ideas or adjustments you believe would better resonate with our organization's values? **Feasibility:** • **Question 11.** From your perspective, what resource allocation or streamlining strategies could make the implementation of these patient navigation for portal enrollment smoother and more efficient? • **Question 12.** Can you recommend any practical measures or adaptations that would simplify the implementation process and overcome any unexpected challenges or barriers?
Sustainment	**Question 13.** It may be easier to get patients to enroll than to ensure they continuously use the patient portal. What strategies or adjustments can we implement to enhance the long-term sustainability of patient navigation for portal enrollment and the continuous use of these portals?
	**Question 14.** Are there any potential risks or barriers you've identified that might pose challenges to the ongoing success of patient navigation for portal enrollment, and how can we address them effectively?

## References

[1] Definitive Healthcare. “How many Federally Qualified Health Centers are there?” (2024). Available online at: https://www.definitivehc.com/blog/how-many-fqhcs-are-there (Accessed June 23, 2025).

[2] Amboree TL Montealegre JR Parker SL Garg A Damgacioglu H Schmeler KM National breast, cervical, and colorectal cancer screening use in federally qualified health centers. JAMA Intern Med. (2024) 184:671–9. 10.1001/jamainternmed.2024.0693 38683574 PMC11059050

[3] Dendere R Slade C Burton-Jones A Sullivan C Staib A Janda M. Patient portals facilitating engagement with inpatient electronic medical records: a systematic review. J Med Internet Res. (2019) 21(4):e12779. 10.2196/12779 30973347 PMC6482406

[4] Matthews AK Steffen AD Akufo J Burke L Diaz H Dodd D Factors associated with uptake of patient portals at a federally qualified health care center. Healthcare. (2024) 12(15):1505. 10.3390/healthcare12151505 39120208 PMC11311389

[5] Herrera MS. “Patient Portal Utilization Effect on Patient Engagement.” (2022).

[6] Ayangunna E. “Factors Associated With Patient Portal Utilization, Preventive Services Utilization, and Health Promoting Behaviors Among Adults in the United States.” (2023).

[7] Coughlin SS Prochaska JJ Williams LB Besenyi GM Heboyan V Goggans DS Patient web portals, disease management, and primary prevention. Risk Manag Healthc Policy. (2017) 10:33–40. 10.2147/RMHP.S130431 28435342 PMC5391175

[8] McCleary NJ Greenberg TL Barysauskas CM Guerette EJ Hassan M Jacobson JO Oncology patient portal enrollment at a comprehensive cancer center: a quality improvement initiative. J Oncol Pract. (2018) 14(8):e451–61. 10.1200/JOP.17.00008 30096276

[9] Matthews AK Watson KS Duangchan C Steffen A Winn R. A study protocol for increasing access to smoking cessation treatments for low-income minority smokers. Front Public Health. (2021) 9:762784. 10.3389/fpubh.2021.762784 34926386 PMC8674302

[10] Erdmann M Edwards B Adewumi MT. Effect of electronic portal messaging with embedded asynchronous care on physician-assisted smoking cessation attempts: a randomized clinical trial. JAMA Netw Open. (2022) 5(2):e220348. 10.1001/jamanetworkopen.2022.0348 35226082 PMC8886534

[11] Carini E Villani L Pezzullo AM Gentili A Barbara A Ricciardi W The impact of digital patient portals on health outcomes, system efficiency, and patient attitudes: updated systematic literature review. J Med Internet Res. (2021) 23(9):e26189. 10.2196/26189 34494966 PMC8459217

[12] Han H-R Gleason KT Sun C-A Miller HN Kang SJ Chow S Using patient portals to improve patient outcomes: systematic review. JMIR Hum Factors. (2019) 6(4):e15038. 10.2196/15038 31855187 PMC6940868

[13] Elkefi S. Disparities and determinants of online medical record access among cancer survivors. Healthcare (Basel). (2024) 12(16):1569. 10.3390/healthcare12161569 PMID: 39201128; PMCID: PMC11353369 39201128 PMC11353369

[14] Short-Russell M Thompson J Waldrop J. Secure messaging: demonstration and enrollment patient portal program: patient portal use in vulnerable populations. Comput Inform Nurs. (2024) 42(2):104–8. 10.1097/CIN.0000000000001098 38206326

[15] Hong YA Jiang S Liu PL. Use of patient portals of electronic health records remains low from 2014 to 2018: results from a national survey and policy implications. Am J Health Promot. (2020) 34(6):677–80. 10.1177/0890117119900591 32030989

[16] Johnson C Richwine C Patel V. Individuals' access and use of patient portals and smartphone health apps, 2020. In: ASTP Health IT Data Brief. Washington, DC: Office of the Assistant Secretary for Technology Policy (2021). p. 1–14. 39571059

[17] DeVoe JE Baez A Angier H Krois L Edlund C Carney PA. Insurance + access ≠ health care: typology of barriers to health care access for low-income families. Ann Fam Med. (2007) 5(6):511–8. 10.1370/afm.748 18025488 PMC2094032

[18] Grossman LV Creber RMM Benda NC Wright D Vawdrey DK Ancker JS. Interventions to increase patient portal use in vulnerable populations: a systematic review. J Am Med Inform Assoc. (2019) 26(8–9):855–70. 10.1093/jamia/ocz023 30958532 PMC6696508

[19] Showell C. Barriers to the use of personal health records by patients: a structured review. PeerJ. (2017) 5:e3268. 10.7717/peerj.3268 28462058 PMC5410160

[20] Chrischilles EA Hourcade JP Doucette W Eichmann D Gryzlak B Lorentzen R Personal health records: a randomized trial of effects on elder medication safety. J Am Med Inform Assoc. (2014) 21(4):679–86. 10.1136/amiajnl-2013-002284 24326536 PMC4078278

[21] Veinot TC Mitchell H Ancker JS. Good intentions are not enough: how informatics interventions can worsen inequality. J Am Med Inform Assoc. (2018) 25(8):1080–8. 10.1093/jamia/ocy052 29788380 PMC7646885

[22] Casey DNP. “The effect of education on portal personal health record use.” (2015).

[23] Greysen SR Harrison JD Rareshide C Magan Y Seghal N Rosenthal J A randomized controlled trial to improve engagement of hospitalized patients with their patient portals. J Am Med Inform Assoc. (2018) 25(12):1626–33. 10.1093/jamia/ocy125 30346543 PMC6289552

[24] Leisy H Ahmad M Guevara G Smith RT. Engaging patients through an iBooks-based patient portal tutorial. BMJ Innov. (2017) 3(3):bmjinnov-2016-000140. 10.1136/bmjinnov-2016-000140

[25] Navaneethan SD Jolly SE Schold JD Arrigain S Nakhoul G Konig V Pragmatic randomized, controlled trial of patient navigators and enhanced personal health records in CKD. Clin J Am Soc Nephrol. (2017) 12(9):1418–27. 10.2215/cjn.02100217 28778854 PMC5586570

[26] Richwine C Johnson C Patel V. Disparities in patient portal access and the role of providers in encouraging access and use. J Am Med Inform Assoc. (2023) 30(2):308–17. 10.1093/jamia/ocac227 36451262 PMC9846679

[27] Matthews AK Steffen AD Burke LA Donenberg G Duangchan C Akufo J The use of navigators to increase patient portal enrollment among patients in a federally qualified health care system. Ethn Dis. (2023) DECIPHeR(Special Issue):117–25. 10.18865/ed.DECIPHeR.117 38846728 PMC11895548

[28] Clinical Directors Network. “Who we are” (2020). Available online at: https://www.CDNetwork.org/ (Accessed June 23, 2025).

[29] Moullin JC Dickson KS Stadnick NA Rabin B Aarons GA. Systematic review of the exploration, preparation, implementation, sustainment (EPIS) framework. Implement Sci. (2019) 14:1–16. 10.1186/s13012-018-0842-6 30611302 PMC6321673

[30] Aarons GA Hurlburt M Horwitz SM. Advancing a conceptual model of evidence-based practice implementation in public service sectors. Adm Policy Ment Health. (2011) 38:4–23. 10.1007/s10488-010-0327-7 21197565 PMC3025110

[31] Braun VC. Thematic analysis. In: Liamputtong P, editor. Handbook of Research Methods in Health Social Sciences. Hoboken, New Jersey: Springer (2016). p. 843–60.

[32] Percac-Lima S Ashburner JM Zai AH Chang Y Oo SA Guimaraes E Patient navigation for comprehensive cancer screening in high-risk patients using a population-based health information technology system: a randomized clinical trial. JAMA Intern Med. (2016) 176(7):930–7. 10.1001/jamainternmed.2016.0841 27273602

[33] Johnson KB Ibrahim SA Rosenbloom ST. Ensuring equitable access to patient portals—closing the “techquity” gap. JAMA Health Forum. (2023) 4(11):e233406. 10.1001/jamahealthforum.2023.3406 37948065 PMC11250922

[34] El-Toukhy S Méndez A Collins S Pérez-Stable EJ. Barriers to patient portal access and use: evidence from the health information national trends survey. J Am Board Fam Med. (2020) 33(6):953–68. 10.3122/jabfm.2020.06.190402 33219074 PMC7849369

[35] Stein JN Klein JW Payne TH Jackson SL Peacock S Oster NV Communicating with vulnerable patient populations: a randomized intervention to teach inpatients to use the electronic patient portal. Appl Clin Inform. (2018) 9(04):875–83. 10.1055/s-0038-1676333 30541152 PMC6291377

[36] Pratt-Chapman ML Silber R Tang J Le PTD. Implementation factors for patient navigation program success: a qualitative study. Implement Sci Commun. (2021) 2:1–9. 10.1186/s43058-021-00248-0 34930503 PMC8685795

[37] Schapira MM Swartz S Ganschow PS Jacobs EA Neuner JM Walker CM Tailoring educational and behavioral interventions to level of health literacy: a systematic review. MDM Policy Pract. (2017) 2(1):2381468317714474. 10.1177/2381468317714474 30288424 PMC6124923

[38] Glaser KM Flores T Lynch M Mossop J Abrams A Johnson C Abstract B105: providing colorectal cancer screening interventions at federally qualified health centers (FQHCs): addressing the issues of language, culture, and health literacy through culturally tailored education and navigation. Cancer Epidemiol Biomarkers Prev. (2020) 29(6_Supplement_1):B105. 10.1158/1538-7755.DISP18-B105

[39] Allen CL Harris JR Hannon PA Parrish AT Hammerback K Craft J Opportunities for improving cancer prevention at federally qualified health centers. J Cancer Educ. (2014) 29:30–7. 10.1007/s13187-013-0535-4 23996232 PMC3920058

[40] Ver Hoeve ES Simon MA Danner SM Washington AJ Coples SD Percac-Lima S Implementing patient navigation programs: considerations and lessons learned from the alliance to advance patient-centered cancer care. Cancer. (2022) 128(14):2806–16. 10.1002/cncr.34251 35579501 PMC9261966

[41] Bush RA Vemulakonda VM Richardson AC Davies SJD Chiang GJ. Providing access: differences in pediatric portal activation begin at patient check-in. Appl Clin Inform. (2019) 10(04):670–8. 10.1055/s-0039-1695792 31509879 PMC6739202

[42] Youn S Geismar HN Pinedo M. Planning and scheduling in healthcare for better care coordination: current understanding, trending topics, and future opportunities. Prod Oper Manag. (2022) 31(12):4407–23. 10.1111/poms.13867

[43] Ramsey A Lanzo E Huston-Paterson H Tomaszewski K Trent M. Increasing patient portal usage: preliminary outcomes from the MyChart genius project. J Adolesc Health. (2018) 62(1):29–35. 10.1016/j.jadohealth.2017.08.029 29169768 PMC5963535

[44] Powell KR. Patient-perceived facilitators of and barriers to electronic portal use: a systematic review. Comput Inform Nurs. (2017) 35(11):565–73. 10.1097/CIN.0000000000000377 28723832

[45] Agboola SO Ju W Elfiky A Kvedar JC Jethwani K. The effect of technology-based interventions on pain, depression, and quality of life in patients with cancer: a systematic review of randomized controlled trials. J Med Internet Res. (2015) 17(3):e65. 10.2196/jmir.4009 25793945 PMC4381812

[46] Renger R. Illustrating the evaluation of system feedback mechanisms using system evaluation theory (SET). Eval J Australas. (2016) 16(4):14–20. 10.1177/1035719X1601600403

